# COVID-19 mortality sentinel surveillance at a tertiary referral hospital in Lusaka, Zambia, 2020–2021

**DOI:** 10.1371/journal.pgph.0003063

**Published:** 2024-03-29

**Authors:** Jonas Z. Hines, Priscilla Kapombe, Adam Mucheleng’anga, Stephen L. Chanda, Amos Hamukale, Mweene Cheelo, Kashala Kamalonga, Leigh Tally, Mwaka Monze, Muzala Kapina, Simon Agolory, Andrew F. Auld, Patrick Lungu, Roma Chilengi

**Affiliations:** 1 U.S. Centers for Disease Control and Prevention, Lusaka, Zambia; 2 Ministry of Health, Lusaka, Zambia; 3 Ministry of Home Affairs, Lusaka, Zambia; 4 Zambia National Public Health Institute, Lusaka, Zambia; 5 University Teaching Hospital, Lusaka, Zambia; GU: Georgetown University, UNITED STATES

## Abstract

Deaths from COVID-19 likely exceeded official statistics in Zambia because of limited testing and incomplete death registration. We describe a sentinel COVID-19 mortality surveillance system in Lusaka, Zambia. We analyzed surveillance data on deceased persons of all ages undergoing verbal autopsy (VA) and COVID-19 testing at the University Teaching Hospital (UTH) mortuary in Lusaka, Zambia, from April 2020 through August 2021. VA was done by surveillance officers for community deaths and in-patient deaths that occurred <48 hours after admission. A standardized questionnaire about the circumstances proximal to death was used, with a probable cause of death assigned by a validated computer algorithm. Nasopharyngeal specimens from deceased persons were tested for COVID-19 using polymerase chain reaction and rapid diagnostic tests. We analyzed the cause of death by COVID-19 test results. Of 12,919 deceased persons at UTH mortuary during the study period, 5,555 (43.0%) had a VA and COVID-19 test postmortem, of which 79.7% were community deaths. Overall, 278 (5.0%) deceased persons tested COVID-19 positive; 7.1% during waves versus 1.4% during nonwave periods. Most (72.3%) deceased persons testing COVID-19 positive reportedly had fever, cough, and/or dyspnea and most (73.5%) reportedly had an antemortem COVID-19 test. Common causes of death for those testing COVID-19 positive included acute cardiac disease (18.3%), respiratory tract infections (16.5%), other types of cardiac diseases (12.9%), and stroke (7.2%). A notable portion of deceased persons at a sentinel site in Lusaka tested COVID-19 positive during waves, supporting the notion that deaths from COVID-19 might have been undercounted in Zambia. Many had displayed classic COVID-19 symptoms and been tested before death yet nevertheless died in the community, potentially indicating strained medical services during waves. The high proportion of cardiovascular diseases deaths might reflect the hypercoagulable state during severe COVID-19. Early supportive treatment and availability of antivirals might lessen future mortality.

## Background

Zambia has recorded >340,000 confirmed COVID-19 cases and >4,000 deaths through July 2023. However, the number of SARS-CoV-2 infections and COVID-19 deaths was likely underestimated [1–4]. Like other countries in Africa, Zambia experienced shortages of COVID-19 testing supplies, especially early in the pandemic [5], making accurate estimates of the COVID-19 pandemic’s impact in Zambia difficult. Moreover, approximately one-third of deaths occur outside health facilities in Zambia, and overall death registration is low in the country [6, 7]. In 2020, less than 20% of expected deaths in the country were registered with the government, varying substantially by province from 0.0% in Northwestern Province to 52.3% in Lusaka Province [6].

Studies of mortality in Zambia during the pandemic indicate undercounting of COVID-19 deaths [8–10]. At the University Teaching Hospital (UTH) in Lusaka, Zambia, Mwananyanda and Gill *et al*. found high (16–32%) COVID-19 test positivity among sampled deceased persons, with a pattern that mirrored COVID-19 waves seen in national surveillance data [8, 9]. Routine mortality surveillance at UTH mortuary identified a similar trend during the first COVID-19 wave, which also corresponded with excess all-cause mortality [10]. A model of COVID-19 seroprevalence and corresponding mortuary data suggested COVID-19 mortality in Lusaka was on par with countries outside of Africa [11].

Assessing the toll of COVID-19 on the country might inform public health and clinical actions for COVID-19 and future potential pandemic threats in Zambia. Additionally, assessing potential surrogate indicators collected as part of routine mortality surveillance could guide future surveillance efforts in places with incomplete death registration systems like Zambia. Here in, we build upon COVID-19 mortality sentinel surveillance in Lusaka, Zambia [12], by linking COVID-19 test and verbal autopsy (VA) results to gain additional insights into COVID-19 mortality in the country. The objectives of this analysis were to 1) describe Zambia’s experience doing mortality sentinel surveillance for COVID-19 in Lusaka, 2) assess how postmortem COVID-19 testing relates to demographics and circumstances of death, VA-assigned cause of death, and antemortem COVID-19 test results, 3) place the findings from this surveillance in a wider country context by applying an age-standardized COVID-19 mortality rate from Lusaka to the population of Zambia, and 4) make recommendations for mortality sentinel surveillance during future outbreaks.

## Methods

### Study design and setting

We analyzed surveillance data of deceased persons of all ages undergoing VA and COVID-19 testing at UTH mortuary in Lusaka, Zambia, from April 2020 through August 2021. In Zambia, approximately two-thirds of deaths occur in health facilities and one-third occur in the community [6]. In health facilities, a medical certification of cause of death (MCCD) form is completed by an attending clinician for deaths that occurred 48 hours or more after admission and do not undergo complete diagnostic autopsy [13]. VA is an alternative to autopsy or an MCCD form when neither option is available. In 25 districts that account for ~50% of Zambia’s population, VA is done for all persons who died in the community and are brought to the local mortuary and for in-patients who died within 48 hours of admission [14].

In Lusaka District, the most populous district in the country, proof of an autopsy, MCCD form, or a VA is required to obtain the burial permit, which itself is required for funerals within the district. For this reason, over 90% deaths are registered in Lusaka District [6], and, because UTH can issue burial permits, its mortuary stores bodies for most community deaths in the district [10, 15]. Thus, Lusaka District, with the highest death registration of all 117 districts in Zambia, was a suitable location to implement sentinel mortality surveillance during the COVID-19 pandemic. Data on in-patient deaths from COVID-19 in Zambia have been previously described [16].

### Data collection

VA entails a standardized questionnaire developed by the World Health Organization that solicits information about the deceased person’s demographics, medical history, symptoms and care proximal to death, place of death, and geographic characteristics of the country/region (VA questionnaire version 2016) [17]. Additionally, questions about COVID-19 exposure, antemortem COVID-19 testing, and diagnosis by a healthcare worker were added to the VA questionnaire in October 2020. VAs were done by trained surveillance officers, who administer the questionnaire to the next-of-kin or a close relative proximal to the time of death. Based on responses to the VA questionnaire, a probable underlying cause of death was assigned by a validated computer algorithm called InterVA5, which is a cause-of-death model that interprets responses to the >300 variables on the WHO 2016 VA questionnaire to assign deaths to all 64 WHO-2016 cause of death categories [18]. The InterVA model processes likelihoods of pregnancy and each cause of death category, outputting up to three causes for each deceased person. COVID-19 questions were truncated from the cause-of-death algorithm and COVID-19 related death was not a possible underlying cause of death assigned by InterVA5. All information in the VA was reported by the respondent and no laboratory testing results were included.

During the COVID-19 pandemic, UTH mortuary did COVID-19 testing on deceased persons of all ages when supplies were available, as part of the country’s COVID-19 surveillance strategy [19]. Trained mortuary attendants performed specimen collection for COVID-19 testing within 24 hours of receipt of the body. A nasopharyngeal specimen was collected from deceased persons using scored swabs inserted into the posterior nasopharynx, while taking appropriate infection prevention measures during specimen collection. The specimen was then transported in viral transport media on ice to the virology lab at UTH where it was tested using the real-time polymerase chain reaction (PCR) test to detect SARS-CoV-2 RNA. In late December 2020, rapid diagnostic tests (RDTs) for SARS-CoV-2 viral antigens were introduced in Zambia, and mortuary attendants began conducting RDTs on deceased persons in early 2021 when supplies allowed. From April 2020 through December 2020, all testing was done by PCR, and from January 2021 through August 2021, testing was a mix of PCR tests and RDTs. COVID-19 test results were recorded in a paper register in the mortuary with the deceased person’s name, date of death, and basic demographic information. Although both PCR tests and RDTs were used at UTH mortuary in 2021, the testing modality was not captured in the testing register. Data from the COVID-19 test register were manually matched to VA data by data clerks based on name, place of residence, sex, age at death, and date of death. Positive COVID-19 test from deceased persons at UTH mortuary were reported to Zambia National Public Health Institute (ZNPHI).

### Study definitions

COVID-19 test positivity was defined as the number of deceased persons testing COVID-19 positive by PCR or RDT divided by the total number of deceased persons who were tested from April 2020 through August 2021. COVID-19 epidemic waves were defined based on visual inspection of an epidemic curve generated using publicly available data, with the predominant SARS-CoV-2 variant driving infections determined by genomic sequencing data from Zambia [16]. A sudden death was defined as dying within 24 hours of being in regular/good health. Classic COVID-19 symptoms were defined as fever, cough, and/or shortness of breath. Throughout this paper, SARS-CoV-2 viral RNA (i.e., PCR) or antigen (i.e., RDT) testing is referred to as COVID-19 testing.

### Data analysis

We analyzed data from deceased persons with both a VA and postmortem COVID-19 test results. First, we analyzed deceased persons’ demographic characteristics and circumstances of proximal to death (i.e., reported symptoms, medical history, care, and place of death), as captured by VA, stratified by their postmortem COVID-19 test results. We used bivariable logistic regression to identify factors associated with testing COVID-19 positive at the time of death (i.e., postmortem). The chi-square test was used to calculate *p* values. Additionally, the monthly postmortem COVID-19 test positivity trend among deceased persons at UTH mortuary was compared to the test positivity for all COVID-19 tests in Zambia [20].

Next, we analyzed underlying probable cause of death for deceased persons as assigned by the InterVA5 model, stratified by their postmortem COVID-19 test results. We also analyzed antemortem COVID-19 test results and health worker COVID-19 diagnosis as reported during VA. Lastly, for deceased persons with antemortem COVID-19 test results, we calculated the sensitivity and specificity of reporting testing COVID-19 positive antemortem using postmortem COVID-19 test positive by PCR or RDT at death as the gold standard comparator.

We estimated the annual age-standardized COVID-19 death rate for Lusaka by multiplying the age-specific COVID-19 positivity by the age-specific deaths from all-causes based on Lusaka mortuary registrations and the Lusaka population projection for 2021 [11]. We then multiplied the age-standardized COVID-19 death rate by the population projection for Zambia in 2021, adjusting for difference in age structure between Lusaka Province and Zambia, to estimate the annual number of COVID-19 deaths and compared this number to officially reported deaths in the country [21]. For this analysis, we assumed SARS-CoV-2 transmission was uniform in Zambia [1], that COVID-19 contributed to the cause of death for 80% of those testing positive [22–24]. and that 10% of deaths were not registered in the Lusaka mortuary registrations data [11].

The study protocol was approved by the ERES Converge IRB and National Health Research Authority in Lusaka, Zambia. This activity was reviewed by CDC and was conducted consistent with applicable federal law and CDC policy (See e.g., 45 C.F.R. part 46.102(l)(2), 21 C.F.R. part 56; 42 U.S.C. §241(d); 5 U.S.C. §552a; 44 U.S.C. §3501 et seq.). All methods were carried out in accordance with relevant guidelines and regulations. This project met requirements for waiver of informed consent documentation, which was granted by ERES Converge IRB in Zambia. Data for this project were accessed on 4/20/2023 and authors did not have access to information that could identify individual participants during or after data collection.

## Results

### Sample size

**Fig 1:**
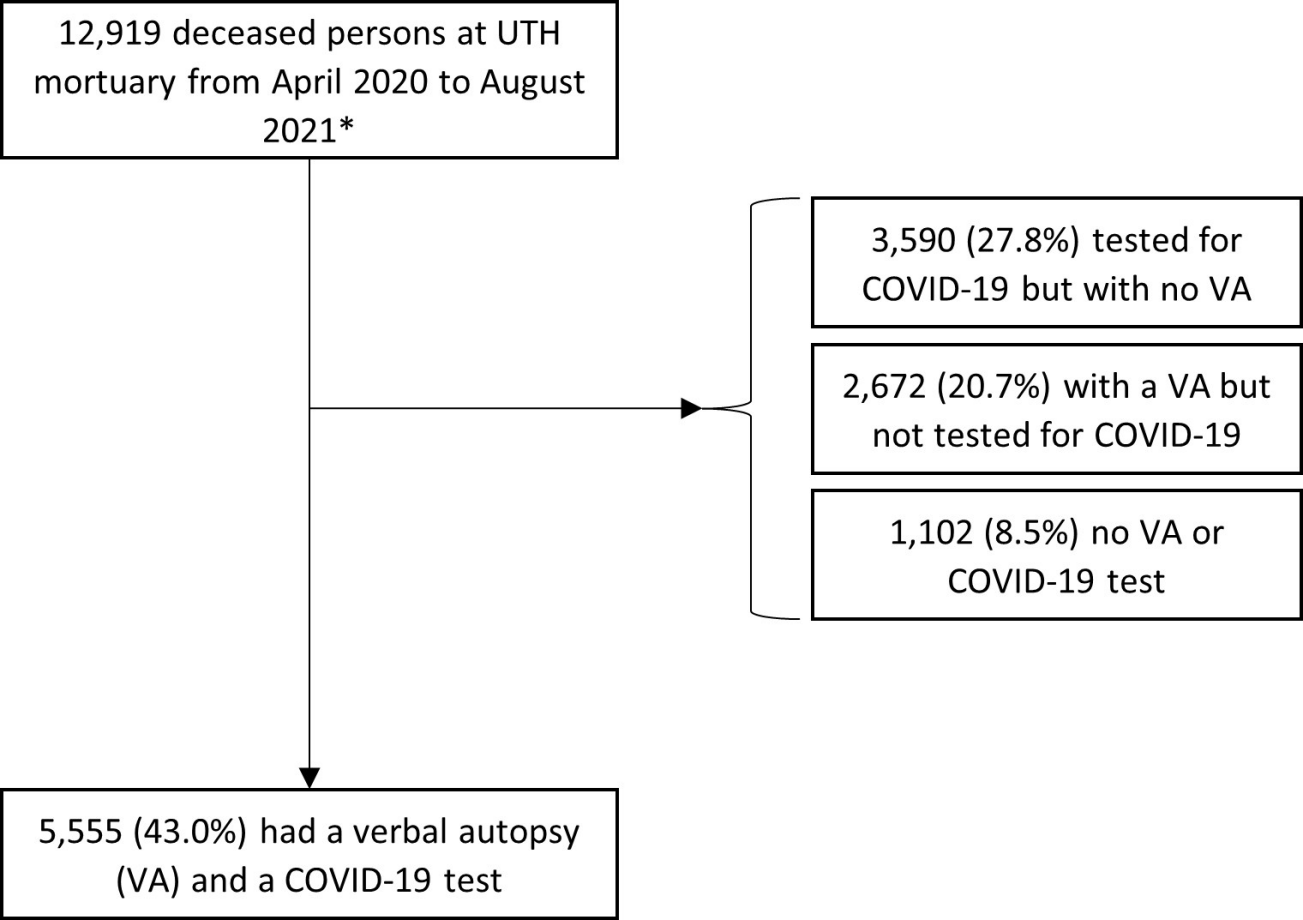
Flow diagram of deceased persons at University Teaching Hospital (UTH) Mortuary April 2020 to August 2021.

There were 12,919 deceased persons brought to UTH mortuary during April 2020 through August 2021 (monthly range 564 [April 2020] to 1,570 [June 2021]). Of these, 9,145 (70.8%) had a PCR or RDT COVID-19 test postmortem and 10,247 (79.3%) had a VA done (Fig 1). Of 9,145 deceased persons who were tested for COVID-19, 3,061 were tested in 2020 and 6,084 in 2021.

### Analysis of deceased persons with postmortem COVID-19 test and VA

In total, 5,555 (43.0%) had both a postmortem COVID-19 test and a VA done during this period. The proportion of deceased persons at UTH mortuary who had a COVID-19 test and VA done varied per month from 2% (April 2020) to 64% (January 2021) (S1 Fig). Some months (e.g., August 2020) had very low numbers of COVID-19 tests done on deceased persons when testing supplies ran low nationally. Approximately four-fifths (79.7%) had died at home. Overall, 59.1% of deceased persons were male and the median age was 46 years (interquartile range: 30–68) (Table 1). Hypertension (34.8%) and HIV (22.2%) were the most common medical morbidities reported.

**Table 1:** Patient characteristics and circumstances of death of deceased persons with a verbal autopsy and postmortem COVID-19 test–Lusaka, Zambia, April 2020 to August 2021.

Variables	Overall, n (%)	Positive COVID-19 test, n (%)	Negative COVID-19 test, n (%)	Odds ratio (95% CI)	*p* value	N missing^¶¶^
	N = 5,555	N = 278	N = 5,277			
Sex					0.876	0
Male	3,282 (59.1)	163 (58.6)	3,119 (59.1)			
Female	2,273 (40.9)	115 (41.4)	2,158 (40.9)	1.02 (0.80, 1.3)		
Age group					< 0.001	0
0–17	765 (13.8)	13 (4.7)	752 (14.3)			
18–49	2,374 (42.7)	87 (31.3)	2,287 (43.3)	2.20 (1.22, 3.96)		
≥50	2,416 (43.5)	178 (64.0)	2,238 (42.4)	4.60 (2.60, 8.13)		
Comorbidities						
HIV positive	1,184 (22.2)	67 (24.5)	1,117 (22.0)	1.15 (0.87, 1.53)	0.334	216
Hypertension	1,700 (34.8)	113 (42.3)	1,587 (34.4)	1.40 (1.09, 1.80)	0.008	674
Cardiac disease	464 (8.4)	35 (12.7)	429 (8.2)	1.63 (1.13, 2.35)	0.009	62
Diabetes mellitus	589 (10.7)	43 (15.6)	546 (10.5)	1.58 (1.13, 2.21)	0.008	58
Asthma	173 (3.1)	9 (3.3)	164 (3.1)	1.04 (0.53, 2.06)	0.912	59
Chronic obstructive pulmonary disease	331 (6.8)	17 (6.3)	314 (6.8)	0.93 (0.56, 1.54)	0.776	664
Chronic kidney disease	161 (2.9)	13 (4.7)	148 (2.8)	1.70 (0.95, 3.03)	0.075	55
Liver disease	193 (3.5)	9 (3.2)	184 (3.5)	0.92 (0.47, 1.82)	0.81	54
Cancer	263 (4.8)	14 (5.1)	249 (4.8)	1.06 (0.61, 1.85)	0.827	55
Tobacco use	1,047 (22.9)	40 (15.9)	1,007 (23.3)	0.62 (0.44, 0.88)	0.007	987
Alcohol use	2,058 (42.2)	75 (28.2)	1,983 (43.0)	0.52 (0.40, 0.68)	< 0.001	677
Place of death					0.926	11
Home	4,421 (79.7)	222 (80.1)	4,199 (79.7)			
Health facility	1,123 (20.3)	55 (19.9)	1,068 (20.3)	0.97 (0.72, 1.32)		
Died during a COVID wave period*	3,511 (63.2)	250 (89.9)	3,261 (61.8)	5.52 (3.72, 8.19)	< 0.001	0
Died suddenly^†^	1,451 (26.1)	61 (21.9)	1,390 (26.4)	0.79 (0.59, 1.05)	0.104	2
Received care before death^‡^	3,724 (67.2)	185 (66.5)	3,539 (67.3)	0.97 (0.75, 1.25)	0.803	16
Tested for COVID-19 antemortem^¶^	1,850 (44.0)	97 (73.5)	1,753 (43.0)	3.68 (2.49, 5.44)	< 0.001	111
Symptoms proximal to death						
Fever	1,902 (34.4)	111 (40.1)	1,791 (34.1)	1.29 (1.01, 1.65)	0.043	32
Cough	1,908 (34.4)	125 (45.0)	1,783 (33.9)	1.60 (1.25, 2.03)	< 0.001	13
Shortness of breath	2,313 (41.8)	142 (51.1)	2,171 (41.3)	1.49 (1.17, 1.89)	0.001	15
Tachypnea	1,195 (21.6)	72 (26.0)	1,123 (21.3)	1.30 (0.98, 1.71)	0.066	10
Chest pain	1,441 (26.8)	88 (32.2)	1,353 (26.5)	1.32 (1.01, 1.71)	0.039	185
Change/loss of taste or smell^¶^	444 (11.9)	38 (29.9)	406 (11.3)	3.37 (2.27, 4.99)	< 0.001	593
Headache	1,614 (30.1)	90 (33.0)	1,524 (30.0)	1.15 (0.89, 1.49)	0.292	196
Vomiting	1,481 (26.7)	55 (19.8)	1,426 (27.1)	0.66 (0.49, 0.9)	0.008	16
Diarrhea	1,093 (19.8)	44 (15.9)	1,049 (20.0)	0.76 (0.55, 1.05)	0.098	21
Abdominal pain	1,334 (24.4)	63 (23.1)	1,271 (24.5)	0.92 (0.69, 1.23)	0.593	95
Rash	115 (2.1)	2 (0.7)	113 (2.1)	0.33 (0.08, 1.34)	0.122	14
Confusion	407 (8.3)	19 (7.2)	388 (8.4)	0.84 (0.52, 1.36)	0.483	669
Classic COVID-19 symptoms**	3,448 (62.1)	201 (72.3)	3,247 (61.5)	1.63 (1.25, 2.13)	< 0.001	0
Asymptomatic^††^	1,179 (21.2)	44 (15.8)	1,135 (21.5)	0.69 (0.49, 0.95)	0.025	0

* Wave period defined as Jun 30 to Sep 21, 2020 (ancestral/wave 1), Jan 3-Mar 19, 2021 (beta variant/wave 2), and May 28-Aug 22, 2021 (delta variant/wave 3).

^†^ A sudden death was defined as dying within 24 hours of being in regular/good health.

^‡^ Indicates person received care for the condition that led to death.

^¶^ COVID-19 questions added to VA in October 2020.

** Defined as fever, cough, or shortness of breath.

^††^ Defined as an absence of any of the following: fever, cough, shortness of breath, tachypnea, chest pain, headache, vomiting, diarrhea, abdominal pain, rash, or mental confusion.

^¶¶^ Number missing deducted from total for each group.

All variables in table derived from verbal autopsy using a standardized questionnaire developed by the World Health Organization administered by trained surveillance officers to the next-of-kin or a close relative proximal to the time of death.

**Fig 2:**
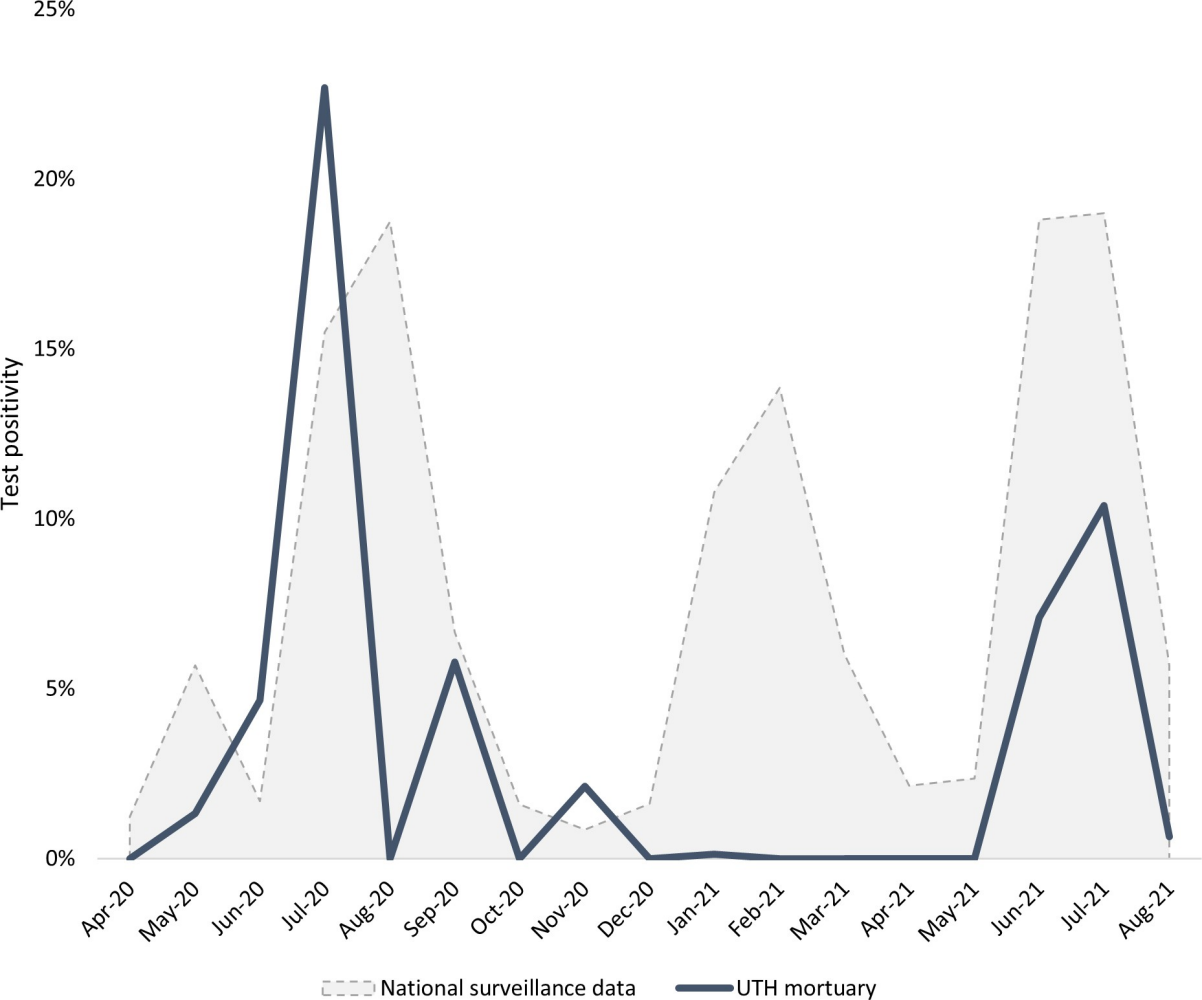
Postmortem COVID-19 test positivity among deceased persons with a verbal autopsy–Lusaka, Zambia, April 2020 to August 2021. Source for national surveillance data: Zambia National Public Health Institute.

Of deceased persons who were tested for COVID-19 and had a VA done, 278 (5.0%) tested COVID-19 positive. COVID-19 test positivity by month varied from 0.0% (multiple months) to 22.7% (July 2020) (Fig 2). Overall, 250 (89.9%) persons died during a COVID-19 wave. COVID-19 positivity was 7.1% during wave periods and was 1.4% during nonwave periods (*p* value <0.01). Two distinct waves were observed in the postmortem COVID-19 test results that corresponded with the first (ancestral strain) and third (delta variant) COVID-19 waves nationally. However, the second (beta variant) COVID-19 wave was not evident in the UTH mortuary COVID-19 testing data (Fig 2). COVID-19 test positivity was 5.0% among persons who died in the community and 4.9% among those who died within 48 hours of admission (*p* value = 0.93) (S1 Table).

Deceased persons testing COVID-19 positive were more likely to be older compared to those testing COVID-19 negative (64.0% ≥50 years-old compared to 42.4%; *p* value <0.01) (Table 1); COVID-19 positivity was 1.7% among deceased persons aged 0–17 years, 3.7% among those 18–49 years, and 7.4% among those ≥50 years [S1 Table] (*p* value <0.01). Additionally, medical comorbidities that were more commonly reported among deceased persons testing COVID-19 positive compared to those testing COVID-19 negative were hypertension (42.3% vs. 34.4%; *p* value <0.01), cardiac disease (12.7% vs. 8.2%; *p* value <0.01), and diabetes mellitus (15.6% vs. 10.5%; *p* value <0.01). Compared to deceased persons testing COVID-19 negative, those testing COVID-19 positive were more likely to had experienced ≥1 classic COVID-19 symptom (72.3% vs. 61.5%; *p* value <0.01), and had been tested for COVID-19 antemortem (73.5% vs. 43.0%; *p* value <0.01).

### Analysis of verbal autopsy cause of death by postmortem COVID-19 test status

Acute (i.e., ischemic) cardiac diseases were the most common underlying cause of death by VA for deceased persons who tested COVID-19 positive and negative (18.3% and 12.2% of deaths, respectively) (Tables 2 and 3). For the 278 deceased persons who tested COVID-19 positive, respiratory tract infections were the second most common cause of death by VA, whereas for the 5,277 that tested COVID-19 negative, respiratory tract infections were the fifth most common (7.8%). Other common causes of death in both groups were other types of cardiac/circulatory system diseases (e.g., heart failure, pericarditis, venous thromboembolic disease), stroke, and HIV related death.

**Table 2:** Probable causes of death among deceased persons testing COVID-19 positive–Lusaka, Zambia, April 2020 to August 2021 (N = 278)*.

Rank	Probable cause of death	n (%)
1	Acute cardiac disease	51 (18.3)
2	Respiratory tract infections/pneumonia	46 (16.5)
3	Other/unspecified cardiac disease	36 (12.9)
4	Stroke	20 (7.2)
5	HIV/AIDS related death	17 (6.1)
6	Pulmonary tuberculosis	16 (5.8)
7	Diabetes mellitus	14 (5.0)
8	Diarrheal diseases	11 (4.0)
9	Digestive neoplasms	8 (2.9)
10	Indeterminate	7 (2.5)

* Underlying cause of death determined by verbal autopsy using WHO 2016 tool. This cause of death is considered probable and is not an official cause of death registered on a death certificate

**Table 3:** Probable causes of death among deceased persons testing COVID-19 negative–Lusaka, Zambia, April 2020 to August 2021 (N = 5,277)*.

Rank	Probable cause of death	n (%)
1	Acute cardiac disease	646 (12.2)
2	HIV/AIDS related death	562 (10.6)
3	Other/unspecified cardiac disease	560 (10.6)
4	Stroke	469 (8.9)
5	Respiratory tract infections/pneumonia	413 (7.8)
6	Diarrheal diseases	288 (5.5)
7	Pulmonary tuberculosis	271 (5.1)
8	Indeterminate	227 (4.3)
9	Digestive neoplasms	222 (4.2)
10	Diabetes mellitus	190 (3.6)

* Underlying cause of death determined by verbal autopsy using WHO 2016 tool. This cause of death is considered probable and is not an official cause of death registered on a death certificate

### Analysis of antemortem COVID-19 test results

Of the 4,317 deceased persons with a VA and COVID-19 testing done from October 2020 through August 2021, 1,850 (42.9%) deceased persons were reportedly tested for COVID-19 prior to death (i.e., antemortem) (S2 Fig); of these, 201 (10.9%) were reportedly COVID-19 positive antemortem. Although antemortem COVID-19 test result information was not collected during the first wave in Zambia, reported antemortem COVID-19 positivity aligned with the second (beta variant) and third (delta variant) waves (S3 Fig). By place of death, 41.4% of persons who died in the community were reportedly tested prior to death compared with 48.5% of persons who died within 48 hours of arriving at a health facility (*p* value <0.01) (S2 Table). Persons who died within 48 hours of arriving at a health facility had greater antemortem COVID-19 positivity than those who died in the community (14.9% vs. 9.6%; *p* value <0.01). The sensitivity and specificity of a COVID-19 positive test antemortem was 66.7% (95% CI: 56.3–76.0) and 91.7% (95% CI: 90.3–93.0) compared to testing COVID-19 positive postmortem by PCR or RDT (S3 Table).

### Age-standardized COVID-19 death rate

The estimated age-standardized COVID-19 death rate per 100,000 population was 219 (95% confidence interval [CI]: 184–254) in 2020 and 211 (95% CI: 175–247) in 2021 (Table 4). This extrapolated to 42,032 (95% CI: 35,260–48,804) COVID-19 deaths in 2020 and 40,486 (95% CI: 33,526–47,446) COVID-19 deaths in 2021.

**Table 4:** Age-standardized death rate from COVID-19 in Lusaka and estimated number of COVID-19 deaths in Zambia.

	2020	2021
Age-standardized COVID-19 death rate per 100,000 population in Lusaka, Zambia*	219 (184–254)	211 (175–247)
Estimated COVID-19 deaths in Zambia^†^	42,032 (35,260–48,804)	40,486 (33,526–47,446)
Officially recorded COVID-19 deaths^‡^	382	3,315

* Calculated from the estimated number of age-specific death registrations in Lusaka (S*ource*: Sheppard R, et al. Nat Commun. 2023;14). The estimate assumes SARS-CoV-2 transmission was uniform in Zambia, that COVID-19 contributed to the cause of death for 80% of those testing positive, and that 10% of deaths were not registered in Lusaka.

^†^ Multiplied age-standardized COVID-19 death rate against the 2010 Zambia Census updated 2021 population projection, adjusting for difference in age structure between Lusaka Province and Zambia.

^‡^ *Source*: Our World in Data COVID-19 data dashboard: https://ourworldindata.org/covid-deaths

## Discussion

A notable portion of deceased persons who died at home or shortly after admission tested COVID-19 positive postmortem during COVID-19 waves in Lusaka, Zambia, and the findings of VA indicate that many likely died from COVID-19-related conditions. If the trend of COVID-19 positivity among deceased persons at UTH was indicative of the national picture, then COVID-19 was the second most common cause of death after HIV in Zambia during this period [6].

This mortality surveillance project was done in a district with high death registration for Zambia. However, because death registration in Zambia is incomplete, had COVID-19 mortality surveillance been more widely implemented during the pandemic, our findings indicate the official count of deaths from COVID-19 would likely had been greater [11]. While our estimate of the COVID-19 death rate in Zambia is greater than one from WHO, it is similar to another widely cited estimate [3, 4]. If other countries in Africa experienced a similar situation during the pandemic, then the continental estimate of reported COVID-19 deaths in Africa of ~175,000 was likely low [25]. Unfortunately, mortality data from COVID-19 has been limited in Africa [8–10, 26, 27], highlighting a need to strengthen mortality surveillance on the continent [28]. Given the substantial number of deaths occurring outside health facilities in Zambia, health facility mortuaries might be a convenient location for mortality sentinel surveillance in future outbreaks. As vital statistics systems are strengthened in Zambia, being able to rapidly implement mortuary-based mortality sentinel surveillance could serve a valuable role in surveillance and case investigation during an unfolding public health threat.

While many deceased persons tested COVID-19 positive during the first and third waves in Zambia, few tested COVID-19 positive during the second (beta variant) wave. While few tested positive postmortem during the second wave, the antemortem testing data in this analysis did correlate with national testing data, as did data from Gill *et al*. [9]. The reasons for this discrepancy are not readily apparent, although COVID-19 positivity in national testing data was also lowest during the beta variant wave [29]. This finding might have been related to the rapid and widespread deployment of COVID-19 RDTs, which are less sensitive than PCR tests, during the middle of the beta variant wave in Zambia. Also, correct application of COVID-19 RDTs by mortuary attendants could have improved over time with more experience.

Only a minority of deceased persons testing COVID-19 positive at death were classified as respiratory tract infections by verbal autopsy. This could reflect the non-specific symptomology of COVID-19, that persons were dying from sequelae of the hypercoagulable state associated with severe COVID-19—noting cardiac diseases and strokes were the other common causes of death in this study [30], or limitations of VA in assigning an accurate cause of death [31, 32]. While VA algorithms for COVID-19 have been developed and performed well at predicting COVID-19 deaths [33], implementation of algorithms with a COVID-19-specific cause of death have been delayed [17]. Our findings indicated that simply relying on a respiratory underlying cause of death from a VA as a surrogate for COVID-19 deaths might underestimate the true burden, demonstrating the value of also measuring all-cause mortality during pandemics [34–36]. Furthermore, differences in antemortem and postmortem COVID-19 test results meant that antemortem positive COVID-19 test was not very sensitive for a positive COVID-19 test at death. This finding could be related to the time between antemortem and postmortem COVID-19 tests, as maximal viral shedding occurs early in infection whereas severe illness and death usually occurs later in the disease course [37, 38]. The findings of VA-coded cause of death and antemortem COVID-19 testing history highlight the challenges of relying on surrogate indicators for surveillance and emphasize the importance of availability of adequate diagnostic tests during outbreaks.

Most persons testing COVID-19 positive postmortem reportedly displayed classic symptoms and were tested before dying yet nevertheless died in the community, potentially pointing to a strained medical system during COVID-19 waves in Zambia. Minchella *et al*. observed increases in the proportion of severe COVID-19 among hospitalized patients within waves in Zambia, postulating this pattern reflected dwindling healthcare capacity as COVID-19 waves progressed [16]. Beyond better access to diagnostic tests, improved availability of antivirals in Zambia could avert mortality during future COVID-19 waves or for other respiratory disease outbreaks like influenza. To address COVID-19’s burden on the health care, Zambia implemented a home-based care model for mild disease and has been an early pilot country in Africa for offering nirmatrelvir/ritonavir [39, 40].

Our findings were similar to a concurrent postmortem study done by Mwananyanda and Gill *et al*. [8, 9], which was unsurprising considering overlap with this surveillance system. However, our data demonstrated a lower percent positivity of deceased persons than Mwananyanda and Gill (i.e., 5.0% vs. 28.1%) as well as a greater proportion of COVID-19 testing prior to death (43.9% vs. 26.8%). Reasons for these differences could reflect different enrollment procedures, different testing approaches (PCR only vs. a mix of PCR and RDTs), or other unaccounted-for bias in either project. This analysis covered a longer period, included data from more deceased persons, and utilized a widely adopted VA tool. Despite the differences, the overarching conclusions are aligned: limited COVID-19 test availability in Zambia likely explains the gap between official statistics and estimated deaths from COVID-19 [3, 4].

There are several limitations to this study. First, COVID-19 test positivity does not establish causality of COVID-19 for the cause of death. No additional pathologic testing was performed to confirm a causal role of SARS-CoV-2, and while a large portion of SARS-CoV-2 infections are asymptomatic [41], a COVID-19 positive test result at death likely reflects such a role [22]. Next, the findings of this study cannot shed light on the proportion of deaths from COVID-19 in the community versus in health facilities because our data did not include testing of all inpatient deaths from COVID-19. Not all deceased persons at UTH were tested for COVID-19 because of an inconsistent supply of testing kits and reagents, and less than half of deceased persons had both a COVID-19 test and VA during the period under review. Furthermore, we could not distinguish between PCR and RDT tests since this information was not recorded in the COVID-19 testing register; this might matter because PCR and RDTs have different diagnostic characteristics—RDTs can have high false negative results, and PCR tests are subject to false positive via cross-contamination [42–45]. Additionally, although respondents were prompted to provide information associated with the deceased person’s final illness, the timing of antemortem COVID-19 testing in the VA questionnaire was general (i.e., a “recent test for COVID-19”). Furthermore, VA responses could be subject to recall and social desirability biases by the next-of-kin completing the questionnaire. Next, VA-coded causes of death categories are broad, and the assignment was not an official cause of death. Lastly, these findings reflect experience from a large tertiary referral hospital in a capital city and the findings might not be generalizable to other parts of Zambia or countries in Africa.

## Conclusions

This project demonstrates the value of mortality sentinel surveillance in a low-income country during a global pandemic. Postmortem testing of primarily community deaths at UTH mortuary demonstrated that deceased persons frequently tested positive for COVID-19 during waves, potentially indicating that deaths from COVID-19 were occurring outside the health system in Zambia and were greater than official statistics. This information was important for the Ministry of Health and ZNPHI to communicate the seriousness of the pandemic to policymakers and provide evidence for implementation of public health and social measures before widespread SARS-CoV-2 exposure and vaccine availability. Additional countries in Africa might publish similar mortality surveillance to provide a better idea of the impact of COVID-19 on the continent. Improving access to diagnostic tests, including supporting regional manufacturing efforts [46], could help alleviate under-ascertainment of deaths in future outbreaks. Additionally, while surrogate indicators can be used for surveillance in the absence of adequate testing, there are potential limitations which might be difficult to understand without further studies. Next, seeing that many COVID-19-positive deceased persons displayed classic symptoms and had been tested prior to dying, strengthening community-based health services during outbreaks could mitigate anticipated strain on health facilities. Sequencing genomes of COVID-19 positive specimens collected from deceased persons could have a role in assessing disease severity when new SARS-CoV-2 variant strains are detected. Strengthening mortality surveillance during outbreaks provided useful insights to inform public health and clinical care to inform decision makers. Further investing in routine mortality surveillance while death registration systems are expanded in Zambia can inform cause of death trend analyses, help monitor for public health events, and serve a role in preparing for potential epidemics.

## Supporting information

S1 Fig Sample size of deceased persons with VA and COVID-19 test by month at University Teaching Hospital mortuary, Lusaka, Zambia, April 2020 to August 2021. “*” Indicate a month when COVID-19 waves were occurring during most days.
(TIF)

S2 Fig Antemortem COVID-19 testing history among deceased persons at University Teaching Hospital mortuary, Lusaka, Zambia, October 2020 to August 2021*. * Questions about antemortem COVID-19 testing and diagnosis by a healthcare worker were added to the standardized verbal autopsy tool in October 2020.
(TIF)

S3 Fig Antemortem COVID-19 test positivity among deceased persons with a verbal autopsy–Lusaka, Zambia, October 2020 to August 2021. (TIF)

S1 Table COVID-19 test positivity of deceased persons with a verbal autopsy and COVID-19 test–Lusaka, Zambia, April 2020 to August 2021. (DOCX)

S2 Table Antemortem COVID-19 testing history according to place of death for deceased persons at University Teaching Hospital mortuary, Lusaka, Zambia, October 2020 to August 2021*. (DOCX)

S3 Table Diagnostic accuracy of COVID-19 test history prior to death for testing COVID-19 positive at death at University Teaching Hospital mortuary, Lusaka, Zambia, October 2020 to August 2021. (DOCX)
