# Supplementary figures and images for: COVID-19 mortality sentinel surveillance at a tertiary referral hospital in Lusaka, Zambia, 2020–2021
Source: PLOS Glob Public Health. 2024 Mar 29;4(3):e0003063. doi: 10.1371/journal.pgph.0003063 (PMC10980196; doi:10.1371/journal.pgph.0003063)

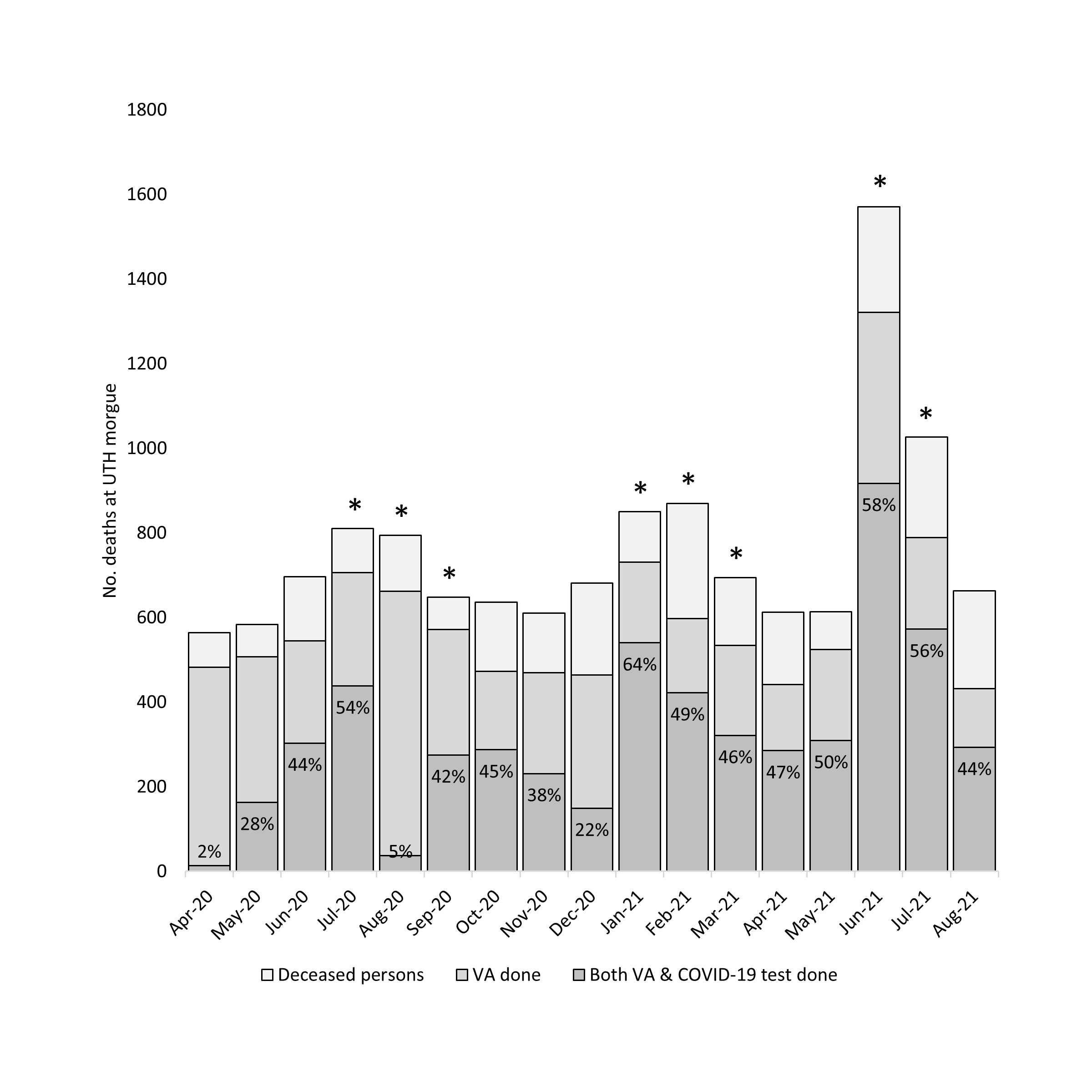

Supplement: S1 Fig — “*” Indicate a month when COVID-19 waves were occurring during most days. (TIF) [file pgph.0003063.s001.tif]

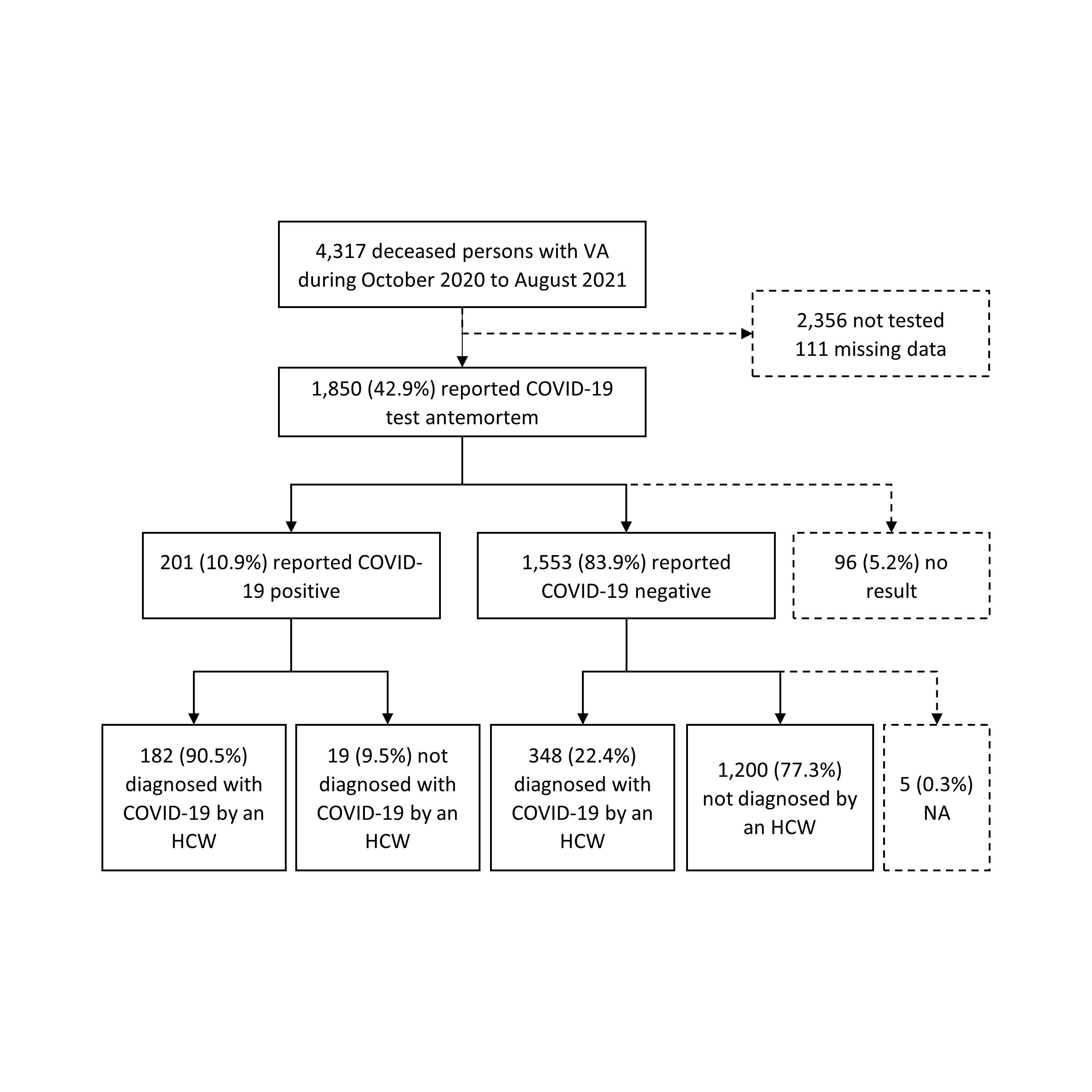

Supplement: S2 Fig — * Questions about antemortem COVID-19 testing and diagnosis by a healthcare worker were added to the standardized verbal autopsy tool in October 2020. (TIF) [file pgph.0003063.s002.tif]

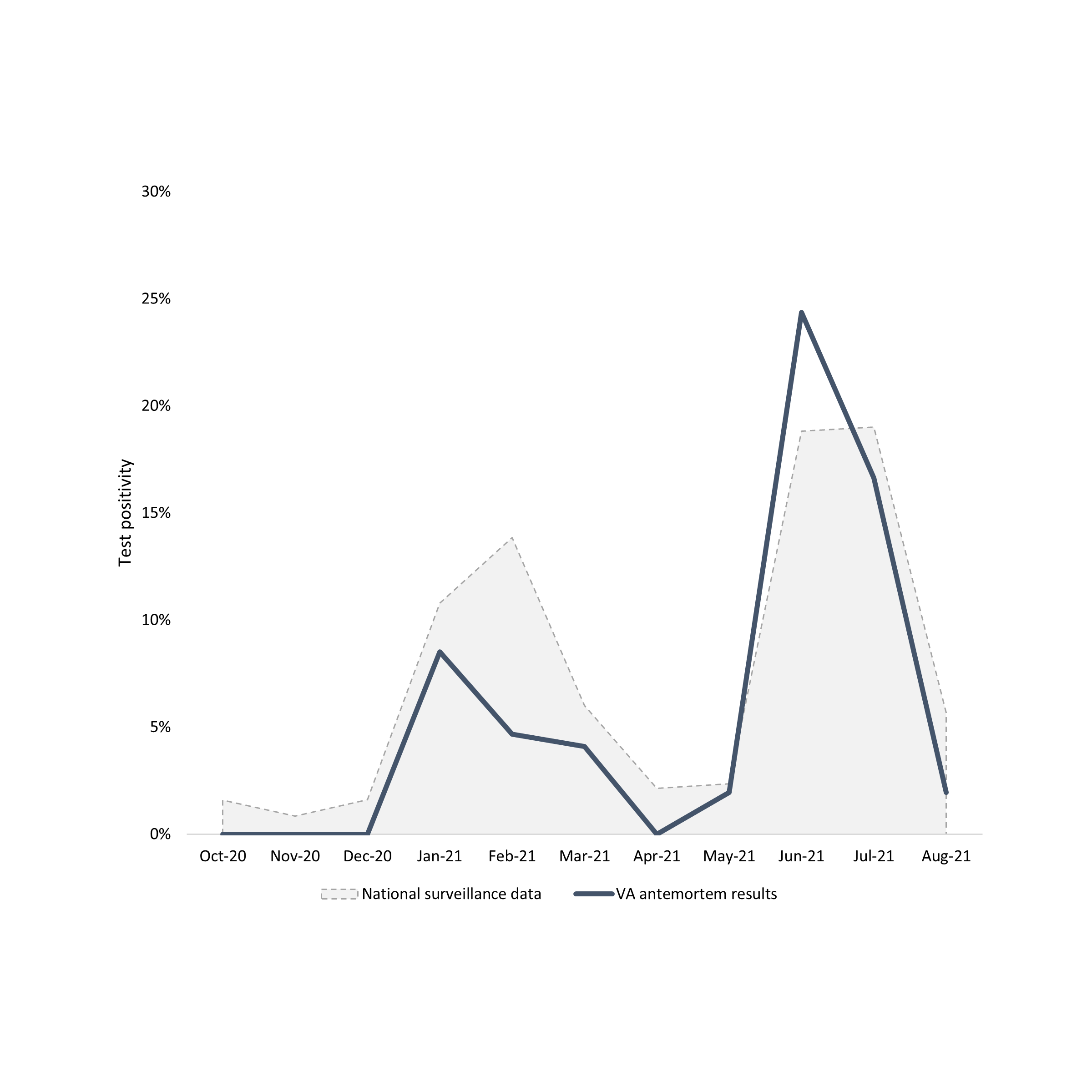

Supplement: S3 Fig — (TIF) [file pgph.0003063.s003.tif]
