# Supplementary material for: COVID-19 mortality sentinel surveillance at a tertiary referral hospital in Lusaka, Zambia, 2020–2021
Source: PLOS Glob Public Health. 2024 Mar 29;4(3):e0003063. doi: 10.1371/journal.pgph.0003063 (PMC10980196; doi:10.1371/journal.pgph.0003063)
Supplement: S1 Table — (DOCX) [file pgph.0003063.s004.docx]

S1 Table. COVID-19 test positivity of deceased persons with a verbal autopsy and COVID-19 test – Lusaka, Zambia, April 2020 to August 2021

| Variable | COVID-19 test  positivity,  n (%) N = 5,555 | *p* value |
| --- | --- | --- |
| Overall | 278 (5.0) |  |
| Sex |  | 0.876 |
| Male | 163 (5.0) |  |
| Female | 115 (5.1) |  |
| Age group |  | <0.001 |
| 0-17 | 13 (1.7) |  |
| 18-49 | 87 (3.7) |  |
| ≥50 | 178 (7.4) |  |
| Place of death* |  | 0.926 |
| Home | 222 (5.0) |  |
| Health facility | 55 (4.9) |  |
| Year |  | <0.001 |
| 2020 | 148 (7.8) |  |
| 2021 | 130 (3.6) |  |
| Died during a COVID wave period^†^ |  | <0.001 |
| Yes | 250 (7.1) |  |
| No | 28 (1.4) |  |
| * For eleven persons the place of the place of death was unknown, including one who tested COVID-19 positive postmortem  ^†^ Wave period defined as Jun 30 to Sep 21, 2020 (ancestral/wave 1), Jan 3-Mar 19, 2021 (beta variant/wave 2), and May 28-Aug 22, 2021 (delta variant/wave 3). | | |
