# Supplementary material for: COVID-19 mortality sentinel surveillance at a tertiary referral hospital in Lusaka, Zambia, 2020–2021
Source: PLOS Glob Public Health. 2024 Mar 29;4(3):e0003063. doi: 10.1371/journal.pgph.0003063 (PMC10980196; doi:10.1371/journal.pgph.0003063)
Supplement: S2 Table — (DOCX) [file pgph.0003063.s005.docx]

S2 Table. Antemortem COVID-19 testing history according to place of death for deceased persons at University Teaching Hospital mortuary, Lusaka, Zambia, October 2020 to August 2021*

|  | Total,  n (%) (N = 4,317)^†^ | Community death,  n (%) (N = 3,434) | In-patient death, n (%) (N = 874)^‡^ | *p* value |
| --- | --- | --- | --- | --- |
| Recently tested for COVID-19 before death | 1,850 (42.9) | 1,421 (41.4) | 424 (48.5) | <0.01 |
| Tested COVID-19 positive | 201 (10.9) | 137 (9.6) | 63 (14.9) | <0.01 |
| * Questions about antemortem COVID-19 testing and diagnosis by a healthcare worker were added to the standardized verbal autopsy tool in October 2020  ^†^ Nine deceased persons did not have place of death captured, including one who reportedly tested COVID-19 positive antemortem  ^‡^ Persons who died at University Teaching Hospital within 48 hours of arrival do not usually have a medical certification of cause of death (MCCD) form, so are eligible for verbal autopsy | | | | |
