# Supplementary material for: COVID-19 mortality sentinel surveillance at a tertiary referral hospital in Lusaka, Zambia, 2020–2021
Source: PLOS Glob Public Health. 2024 Mar 29;4(3):e0003063. doi: 10.1371/journal.pgph.0003063 (PMC10980196; doi:10.1371/journal.pgph.0003063)
Supplement: S3 Table — (DOCX) [file pgph.0003063.s006.docx]

S3 Table. Diagnostic accuracy of COVID-19 test history prior to death for testing COVID-19 positive at death at University Teaching Hospital mortuary, Lusaka, Zambia, October 2020 to August 2021

|  | | | COVID-19 test postmortem | |
| --- | --- | --- | --- | --- |
|  |  |  | (+) | (-) |
| COVID-19 test antemortem* | (+) | | 64 | 137 |
|  | (-) | | 32 | 1,521 |
| Sensitivity | | 0.667 (0.563, 0.760) | | |
| Specificity | | 0.917 (0.903, 0.930) | | |
| Positive predictive value | | 0.318 (0.255, 0.388) | | |
| Negative predictive value | | 0.979 (0.971, 0.986) | | |
| * Timing of COVID-19 test was not specified in questionnaire. Specific question is “Did s(h)e have a recent test for COVID-19?” in the section on the medical history associated with the person’s final illness. | | | | |
